# Performance of the Aptis distal radioulnar joint implant: kinematic and geometric analysis

**DOI:** 10.1177/17531934241274142

**Published:** 2024-10-11

**Authors:** Joris G. M. Oonk, Shirley D. Stougie, Johannes G. G. Dobbe, Marco J. P. F. Ritt, J. Henk Coert, Geert J. Streekstra

**Affiliations:** 1 Amsterdam UMC location University of Amsterdam, Biomedical Engineering and Physics, Amsterdam, the Netherlands; 2 Amsterdam Movement Sciences, Musculoskeletal Health - Restoration and Development, Amsterdam, the Netherlands; 3 Amsterdam UMC location University of Amsterdam, Plastic, Reconstructive and Hand surgery, Amsterdam, the Netherlands; 4 University Medical Center Utrecht, Plastic, Reconstructive and Hand Surgery, Utrecht, The Netherlands

**Keywords:** 4-D computed tomography, distal radioulnar joint, kinematics, geometry, bilateral, radius, ulna, implant, arthroplasty

## Abstract

This study reviews the performance of the Aptis distal radioulnar joint arthroplasty by comparing multiple kinematic and geometric measurements in the operated and contralateral healthy forearm to elucidate whether these are altered after arthroplasty. Forearm geometry and motion were captured using 3-D and 4-D computed tomography in 12 patients with unilateral Aptis arthroplasties. After segmentation and registration, the axis of forearm rotation, translation of the radius along the ulna and range of wrist flexion-extension were measured, and the Dice coefficient and Hausdorff distance were calculated. The forearm rotation axis in the corrected arm deviated 2.3° from the healthy contralateral rotation axis, radial translation along the ulna decreased by 45% and wrist flexion-extension also decreased significantly. Multiple intra-individual geometric differences were observed. The Aptis distal radioulnar joint arthroplasty considerably alters forearm kinematics, which can have clinical implications.

**Level of evidence:** IV

## Introduction

Arthroplasty of the distal radioulnar joint (DRUJ) is mainly indicated when the joint is affected by osteoarthritis and instability or after a failed ulnar head resection procedure (Kakar et al., 2014; Warwick et al., 2013). DRUJ arthroplasty is categorized into three types (Calcagni and Giesen, 2016). A Type I arthroplasty interposes allo- or autograft material between the articular surfaces of the radius and ulna to restore joint function (Bain et al., 1995; Papatheodorou et al., 2013). In Type II, the ulnar head is partially or fully replaced (Garcia-Elias, 2007; Willis et al., 2007). A Type III arthroplasty replaces the ulnar head and the articular surface of the radius and mimics the function of the triangular fibrocartilage complex (TFCC), creating a semi-constrained joint (Kakar et al., 2014; Scheker, 2008).

Currently, the Aptis DRUJ implant (Aptis Medical, Glenview, KY, USA) is the only available Type III implant. Patient satisfaction is high (Bellevue et al., 2017; Calcagni and Giesen, 2016; Kakar et al., 2014) and function generally improves after the procedure (Brannan et al., 2022; DeGeorge et al., 2017; Kakar et al., 2014; Lans et al., 2019). However, complications occur in 28%–44% of patients (Bellevue et al., 2017; Brannan et al., 2022; DeGeorge et al., 2017; Moulton and Giddins, 2017) and revision surgery is common (Bellevue et al., 2017; Brannan et al., 2022; Lambrecht et al., 2022; Pääkkönen, 2022). The discrepancy between functional outcomes and the occurrence of complications and the need for revision raises the question whether the current functional and patient-reported outcomes are suitable for assessing the performance of the implant. Although it is standard practice to compare the preoperative and postoperative states in the same forearm, it might be better to compare the postoperative forearm with the healthy contralateral arm. Since the aim is to restore the original function, it could be more valuable to use measurements that quantify differences in geometry and kinematics. The Dice coefficient (Dice, 1945) can quantify the geometric agreement between the shape of the radius on the operated side and that of the healthy radius by measuring the percentage of overlapping volume in two 3-D models. Comparison of the axes of forearm rotation in the operated and contralateral arms could serve to quantify the success of kinematic restoration.

In this study, we have quantified the performance of the Aptis DRUJ implant by comparing several geometric and kinematic measurements with the healthy contralateral DRUJ, using three- and four-dimensional computed tomography (3-D CT, 4-D CT).

## Methods

A total of 12 patients (eight men, four women; mean age 51 years; age range 26–65 years) with a mean follow-up of 56 months (range 13–106) were included. We included patients aged over 18 years who had undergone unilateral Aptis DRUJ arthroplasties at our hospital more than 6 months before the start of this study and who were willing to give informed consent and did not have any ongoing complications related to the implant. The Exclusion criteria were a history of trauma, injury or other damage to the contralateral forearm, pregnancy and the inability to understand or give informed consent. All included patients had a history of a distal radial fracture in the forearm with the implant.

### Image acquisition

**Figure 1.**
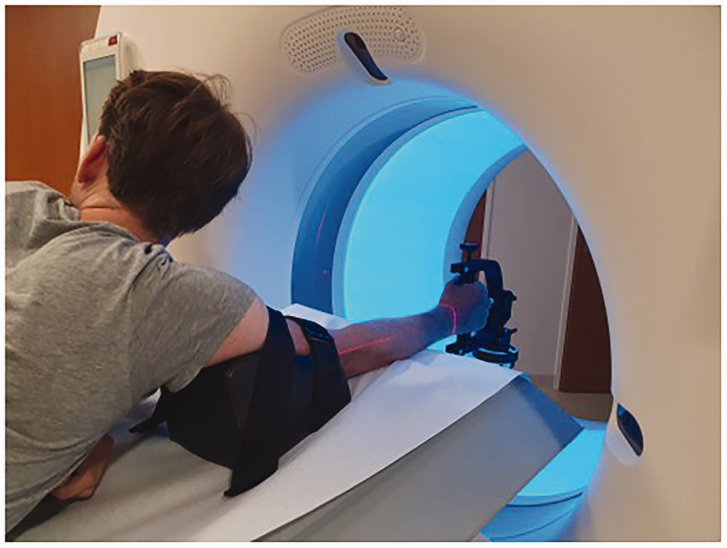
Patient positioning and hand guiding device for forearm rotation during 4-D CT.

A Somatom Force CT scanner (Siemens Healthineers AG, Erlangen, Germany) was used to acquire spiral 3-D CT scans (120 kV, 65 mAs) of both forearms. To find the forearm rotation axis, each arm was 4-D CT scanned (120 kV, 15 mAs; 33 frames) in the proximal radioulnar joint (PRUJ) region and in the DRUJ region. Because metal artifacts significantly disturbed the DRUJ images in the affected arm, this arm was scanned at the mid-shaft region instead of the distal region. Both wrists were also 4-D CT scanned during flexion-extension (FE) motion. The patients were scanned in the prone position with one arm overhead and the elbow in approximately 30° of flexion. Two custom-built devices, one designed for forearm rotation and the other for wrist flexion and extension, were sequentially mounted to the scanner table. These devices provided a stable grip for the patient and guided the hand during forearm rotation (Figure 1) and wrist FE motion, respectively. They also ensured that the distal radioulnar joint or wrist remained within the field of view of the CT scanner.

Before the 4-D CT acquisition, the patients were given instructions and practised forearm rotation and wrist FE motion until they were comfortable with the procedure. Forearm rotation took 20 seconds, starting at a fully supinated position and moving to a fully pronated position. Wrist FE motion took 10 seconds and started at a fully extended position, finishing at a fully flexed position. While scanning, the motion was visually checked and a countdown was provided over the scanner intercom.

### Image processing

Custom software was used for image analysis (Dobbe et al., 2019). In brief, the images of the left arm were mirrored to the right arm to facilitate comparison of both forearms during quantitative analysis. 3-D models of the radius, ulna and capitate were generated from the images obtained from the 3-D CT scan using a process called segmentation. The capitate was segmented as its position with respect to the radius was chosen to define the position of the wrist (FE angle) in this study. The 3-D models of the radius and ulna were then clipped (approximately 57 mm, corresponding to the size of the 4-D CT images along the gantry axis) into distal and proximal sections. Finally, these clipped sections of the radius and ulna models and the capitate model were aligned to the corresponding 4-D CT images through a process called registration. This resulted in 3-D representations of the motion of the described bone models during forearm rotation and wrist FE motion. The methodological error of the employed 4-D CT scanning protocol and image processing were approximately 0.08 mm and 0.1° for translational and rotational errors respectively (Oonk et al., 2023).

### Rotation axis

The axis of rotation in the healthy forearm was determined using a circle-fit algorithm using the following steps. First, the centroid coordinates of a distal and proximal segment of the radius were determined in the 4-D CT frames in which the respective segments were present. Then planes were fitted to both the distal and proximal group of coordinates. After projection of the distal and proximal coordinates on to their respective planes, a circle was fitted to the in-plane 2-D coordinates for both planes, resulting in two circles (Oonk et al., 2023). The centre coordinates of these circles defined the forearm rotation axis.

In the forearms with an Aptis DRUJ implant, the centroid of the plastic sphere in the radial component was selected as the distal endpoint of the forearm rotation axis. Unfortunately, the centroid position of the plastic sphere could not easily be determined from the CT scans owing to metal artifacts. Therefore, computer-aided design (CAD) models of the Aptis implant (size 10 and 20) were created, using Autodesk Fusion 360 (Autodesk, San Francisco, CA, USA). The CAD 3-D model of the implant corresponding to the implant size of the patient was aligned with the radial component segmented from the 3-D CT scan, using iterative closest point (ICP) registration (Besl and McKay, 1992). This permitted quantification of the centroid coordinate of the CAD model’s plastic sphere and the distal endpoint of the forearm rotation axis.

**Figure 2.**
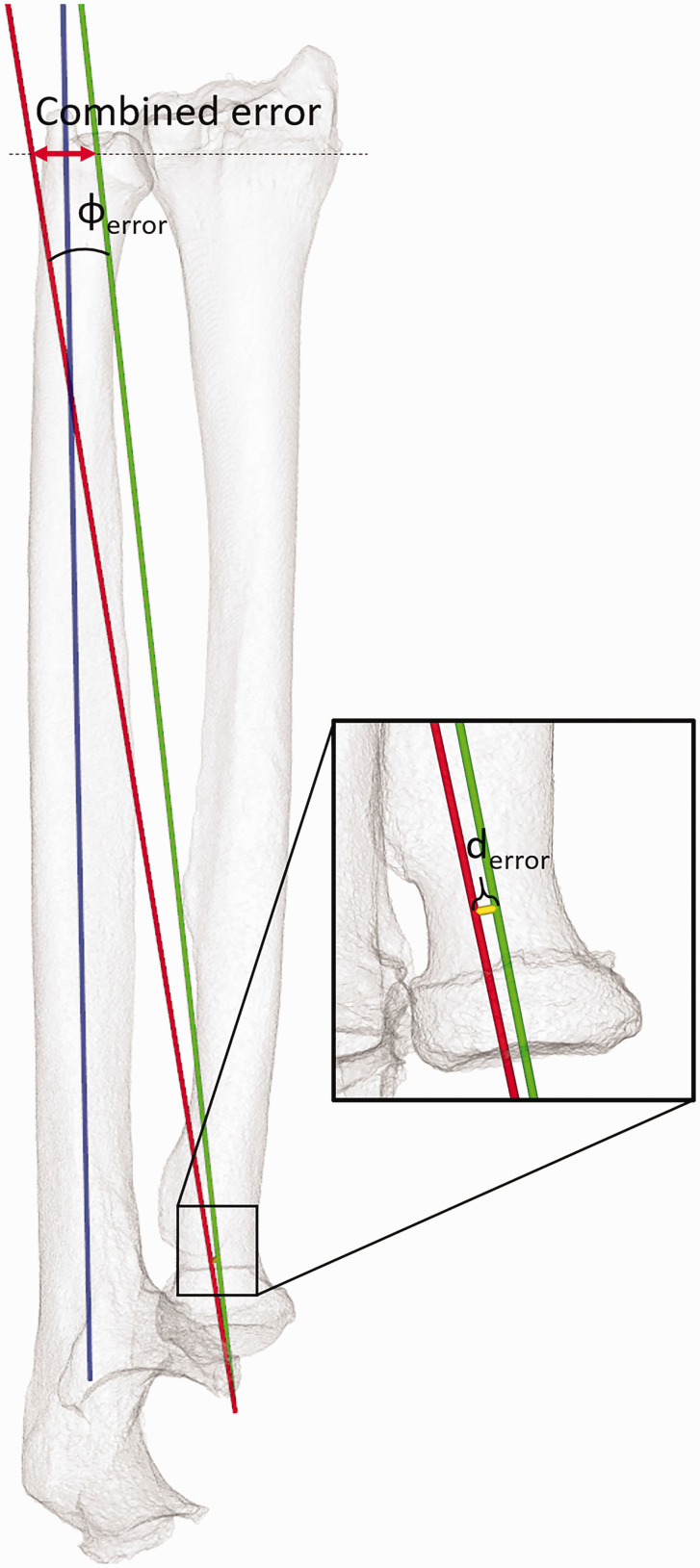
Parameters for quantifying malpositioning of the forearm rotation axis (red =  axis with implant; green =healthy forearm), showing the angular error (φ_error_) and position error (d_error_). The combined error is found as the distance between the points where these axes intersect a plane at the distal radioulnar level (dotted line). The blue axis defines the ulnar axis along which the radius translates during forearm rotation.

For comparison of both rotation axes, the ulnae of both forearms (mirrored left and right) were aligned using ICP registration (Besl and McKay, 1992). The rotation axes determined previously were fixed to their respective ulnae, allowing for a comparison after successful registration. The angle between the rotation axes represented the angular error (φ_error_) (Figure 2). The positional error was defined by the shortest distance between both rotation axes (d_error_) (Figure 2). The angular and positional errors were also combined to quantify overall malpositioning of the forearm rotation axis when using the Aptis implant (Figure 2). To this end, a plane was placed at the DRUJ level where the radius is closest to the ulna. The plane normal (the perpendicular axis originating from the centre of the plane) was aligned with the gravitation axis, also known as the major axis of inertia, of the radius. The combined error was expressed as the Euclidean distance between the points where the rotation axes intersected the plane.

### Radial translation along the ulnar axis

The radius translates along the longitudinal axis of inertia of the ulna (ulnar axis) during forearm rotation (Oonk et al., 2023). This translation was quantified by tracking translation of the sigmoid notch centroid (for healthy arms), or the centroid of the plastic sphere in the Aptis implants (for operated arms), along the ulnar axis during forearm rotation. The translation was subsequently plotted against the forearm angle of rotation and a second order polynomial was fitted through the data points. The range of the polynomial defined the translation of the radius along the ulnar axis.

### Flexion-extension range of motion

To calculate FE range of motion (ROM), the finite helical axis (FHA) of the capitate relative to the radius was used as it approximates motion of the hand during FE. The FHA describes a single axis in 3-D space along which a body translates and around which it rotates. The FHA and rotation magnitude were computed from the transformation matrix of capitate motion between the extreme frames of the 4-D CT scan capturing wrist FE.

### Geometric correspondence

**Figure 3.**
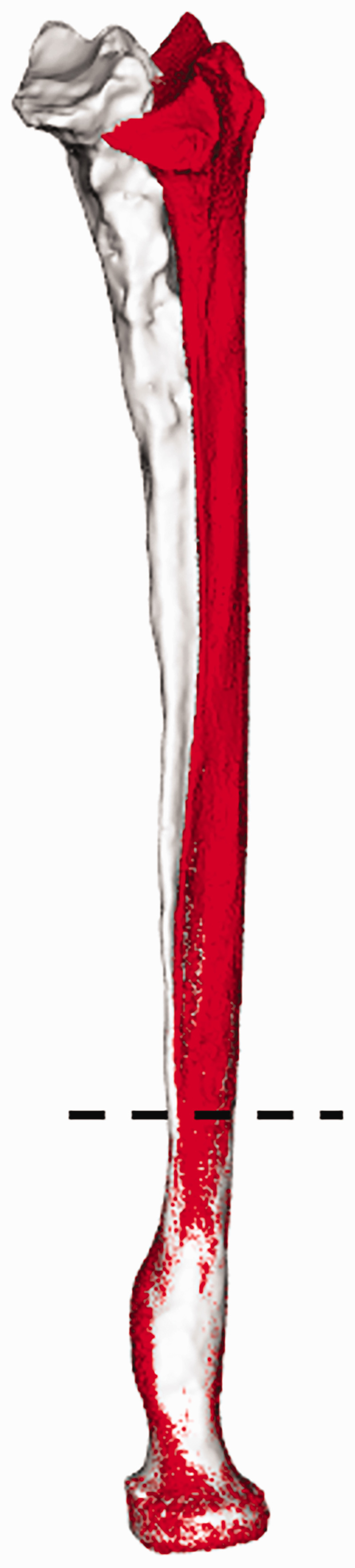
Alignment of the affected radius with the healthy radius. The proximal part (30%, below the dashed line) of the radius unaffected by distal injury is registered to the healthy radius. After registration, the distal part of the affected radius was linked to the proximal part to visualize the effect of deformation and remodelling.

Radius geometry was compared by calculating the Dice coefficient (Dice, 1945) and the Hausdorff distance (Huttenlocher et al., 1993). The Dice coefficient quantifies the degree of volume overlap between two 3-D models. The Hausdorff distance is the longest distance between any point on one 3-D model and the nearest point on the other 3-D model. Quantification required ICP registration (Besl and McKay, 1992) to align the proximal radius on the operated side to the healthy radius. In this study, the proximal 30% of the operated radius was registered to the healthy radius (Figure 3), thereby excluding the region affected by the initial fracture, malunion or remodelling as a result of the implant from the registration procedure, but still ensuring accurate registration. After alignment, the radius models were exported to 3D-slicer (https://www.slicer.org/) to calculate the Dice coefficient and Hausdorff distance.

### Statistical analysis

Originally, all samples were tested for normality using a Shapiro–Wilk test (Table 1; non-normal indicated by ^a^). Several statistical tests (Table 1) were carried out to assess significant differences in performance between the corrected arm and the healthy contralateral arm, and to find potential correlations between two metrics. A correlation could unveil possible causes for differences found in forearm kinematics. All tests were done using Python (van Rossum and Drake, 2009) supplemented with the SciPy stats package (Virtanen et al., 2020). The level of significance (α) was set at 0.05.

**Table 1.** Summary of parameters, statistical tests used and the goals of each test.

Parameter 1	Parameter 2	Test	Goal
Angular error axis of rotation	Dice coefficient^a^	Spearman	Find correlation
Angular error axis of rotation	Hausdorff distance	Pearson	Find correlation
Δ Translation^a^	Dice coefficient^a^	Spearman	Find correlation
Δ Translation^a^	Hausdorff distance	Spearman	Find correlation
Translation healthy	Translation Aptis implant^a^	Wilcoxon signed rank	Test difference
Δ Translation^a^	Δ range of pronation supination	Spearman	Find correlation
Range of wrist flexion-extension Aptis implant	Range of wrist flexion-extension healthy	Paired *t*-test	Test difference

a Non-normally distributed sample.

Δ: change in.

## Results

### Rotation axis

**Figure 4.**
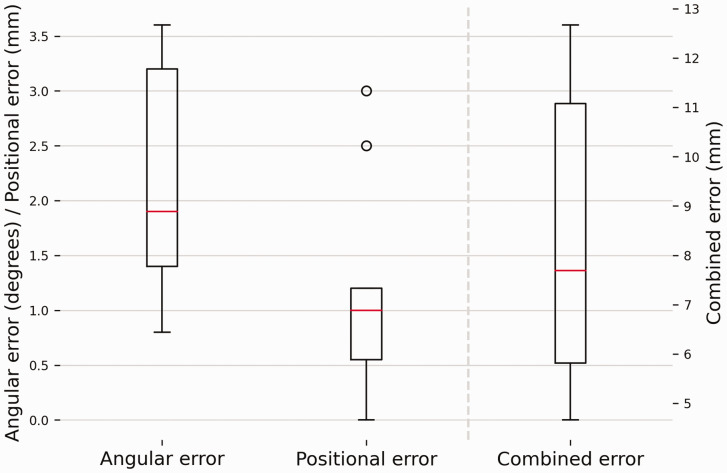
Deviations in the forearm rotation axis with respect to the healthy contralateral forearm. The box plots show the interquartile range and median (red horizontal line), the biggest and smallest datapoints that are not considered to be outliers (<1.5 IQR from the box) are represented by the whiskers. Outliers are indicated by black circles. A visual representation of the errors shown in this figure can be found in Figure 2.

The mean rotation axis of the corrected forearm deviated 2.3° (standard deviation [SD] 0.9) from the rotation axis of the healthy forearm. The mean positional error was 1.0 mm (SD 0.9). Both errors contributed to a mean combined error of 8.2 mm (SD 2.9) at the DRUJ level (Figure 4). No significant correlations were found between the angular error and the two geometric parameters.

### Radial translation along the ulnar axis

**Figure 5.**
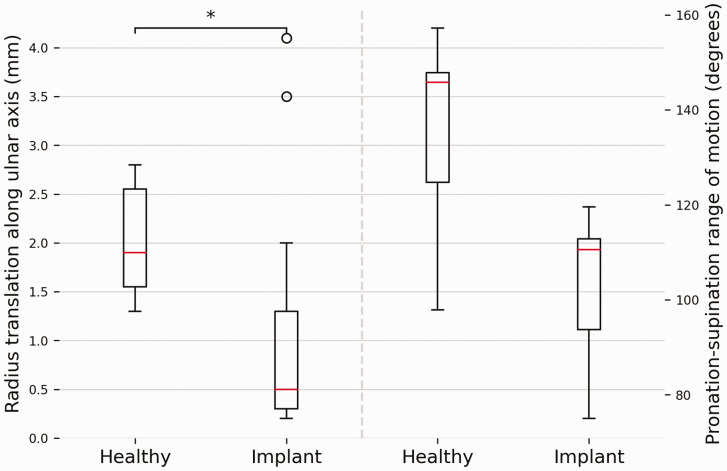
A comparison between forearms with and without Aptis implant regarding translation along the ulnar axis and pronation-supination range of motion. The box plots show the interquartile range and median (red horizontal line), the biggest and smallest datapoints that are not considered to be outliers (<1.5 IQR from the box) are represented by the whiskers. Outliers are indicated by black circles.

**Figure 6.**
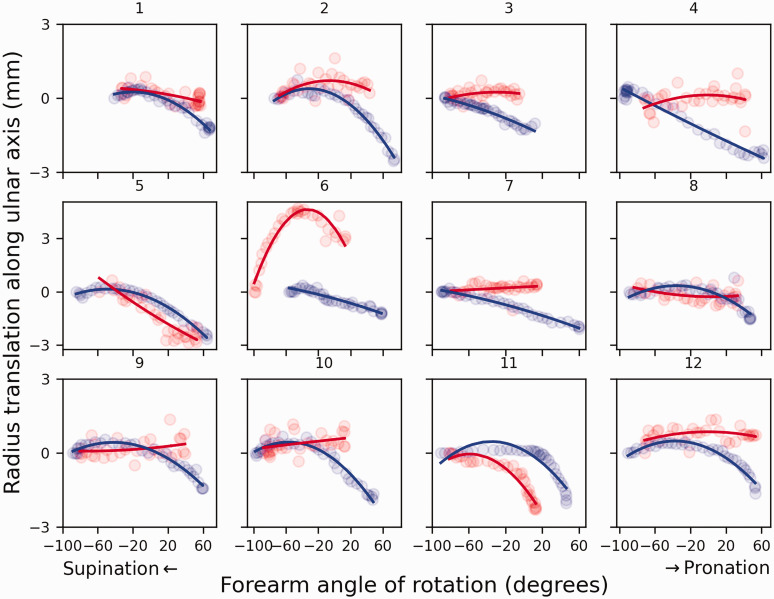
Translation of the radius along the ulnar axis in 12 patients with an Aptis implant (red) compared to the contralateral healthy side (blue).

Grouped results for translation of the radius along the ulnar axis during forearm rotation are shown in Figure 5 and a case-by-case visualization is given in Figure 6. The translation along the ulnar axis in the healthy arms was 2.0 mm (SD 0.6). For the affected arms, the median translation was 0.8 mm (interquartile range [IQR] 0.75–0.84). The median forearm rotation ROM for healthy arms was 144.5° (IQR 130.1–147.6). The mean rotation for arms with the implant was 104.4° (SD 13.8). Translation along the ulnar axis was found to differ significantly between healthy and corrected forearms (*p* = 0.03).

No correlation was found between the decrease in translation along the ulnar axis and the decrease in forearm rotation ROM, Dice coefficient or Hausdorff distance.

### Flexion-extension range of motion

**Figure 7.**
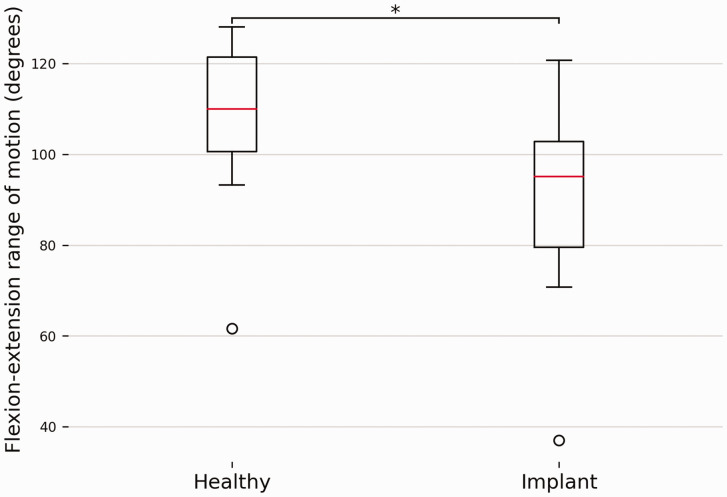
A comparison of wrist flexion-extension range of motion between forearms with and without an Aptis implant. The box plots show the interquartile range and median (red horizontal line), the biggest and smallest datapoints that are not considered to be outliers (<1.5 IQR from the box) are represented by the whiskers. Outliers are indicated by black circles.

Mean wrist FE ROM was 105.3° (SD 18.6) in healthy forearms, and decreased significantly (*p* = 0.0003) to 87.6° (SD 22.1) in arms with an Aptis implant (Figure 7).

### Geometric correspondence

**Figure 8.**
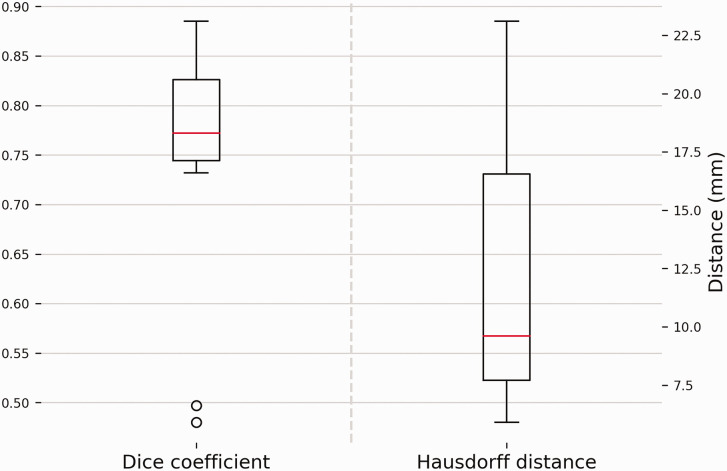
Geometric correspondence between radius bones represented by the Dice score and Hausdorff distance. A higher Dice score is equivalent to an increased amount of volumetric overlap and thus better correspondence. A large Hausdorff distance equates to a large distance between the bone models in a specific area and thus poor correspondence. The box plots show the interquartile range and median (red horizontal line), the biggest and smallest datapoints that are not considered to be outliers (<1.5 IQR from the box) are represented by the whiskers. Outliers are indicated by black circles.

The median Dice coefficient and the mean Hausdorff distance were 0.45 (IQR 0.30–0.95) and 12.2 mm (SD 5.6), respectively (Figure 8). No significant correlations were found between these geometric parameters and other kinematic parameters.

## Discussion

This study has shown that the Aptis DRUJ implant alters forearm kinematics. Deviation of the forearm rotation axis and a reduced translation of the radius along the ulnar axis compared to the contralateral side were the main observations. The forearm rotation axis in arms with the Aptis implant deviated 2.3° with the distal end towards the ulna, compared to the healthy arm. Translation of the distal radius along the ulnar axis in the corrected arm decreased by 45% relative to the healthy arm. These differences may seem small but they resulted in a new centre of rotation at the level of the DRUJ that was 8.2 mm away from where it was originally located. This may lead to different stress patterns in the bones or the implant, possibly resulting in complications or even the need for revision surgery.

**Figure 9.**
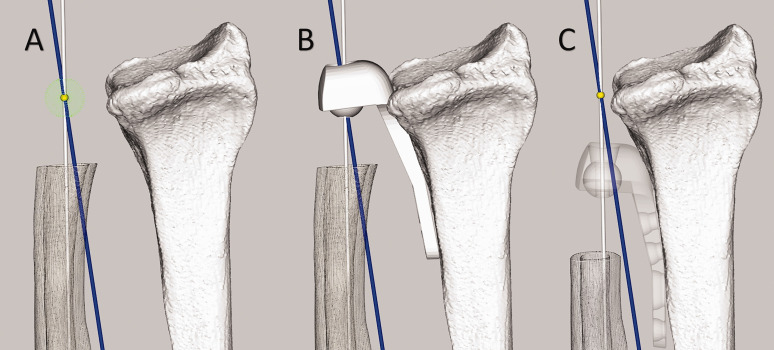
Implant placement and the effect on the rotation axis. The ulnar medullary axis is shown in white, the optimal forearm rotation axis in blue and the intersection point of the two axes in yellow. (a) Required position of the Aptis implant plastic sphere (green) to restore the forearm rotation axis of the healthy contralateral side. (b) Required placement of radial component of Aptis implant to recreate healthy rotation axis. Note the carpal interference. (c) More proximal placement of the Aptis implant in agreement with the surgical manual. Note that the radius is pulled towards the ulna and that the centre of the plastic sphere does not intersect the blue rotation axis, thus forcing a different rotation axis.

Restoring the normal anatomical rotation axis of the forearm could prevent issues related to the Aptis implant. Unfortunately, perfectly restoring the forearm rotation axis is not feasible since the axis is dictated by the location of the polyethylene sphere in the radial component and ulnar stem placement in the ulnar medullary cavity. In healthy forearms, the axis of rotation intersects the ulnar fovea and the centre of the radial head (Matsuki et al., 2010; Nakamura et al., 1999; Oonk et al., 2023; Tay et al., 2010). In an Aptis implant, placing the centre of the implant’s polyethylene sphere exactly at the intersection of the rotation axis and the centreline of the medullary cavity would result in the desired rotation axis (Figure 9(a)). However, this would lead to interference of the implant with the radiocarpal joint (Figure 9(b)). According to the Aptis surgical manual, the distal end of the radial trial plate should be positioned 3–20 mm below the lunate fossa (Aptis Medical, 2019) to avoid carpal impingement. In both instances, the rotational centre is created more proximal and lateral to the rotational centre of the healthy forearm, causing the radius and the rotation axis to be drawn towards the ulna (Figure 9(c)).

Various studies (Fu et al., 2009; Kazama et al., 2016) have reported the same magnitude of translation of the radius along the ulnar axis in healthy forearms as was found for healthy forearms in this study. In contrast, the mean translation of the radius in the corrected DRUJ was only 1.1 mm and statistically different. Three patients (patients 5, 6 and 11) showed similar or increased translation relative to the healthy contralateral DRUJ, whereas the other patients showed almost no translation (Figure 6). Figure 6 and the statistical analysis show that the decrease cannot be attributed to a decreased range of forearm rotation since the profile is markedly different and no correlation was found. It should be noted that elbow position affects radial translation along the ulna (Fu et al., 2009). However, since the scans in this study were made at the same elbow angles, the assessments of the intraindividual differences were not affected. The cause of the limited translation of the radius remains to be determined, but interference of the polyethylene sphere with the ulnar stem, soft tissue or scar tissue are plausible explanations.

The range of FE ROM has been shown to improve after DRUJ arthroplasty (Bizimungu and Dodds, 2013; DeGeorge et al., 2017; Kachooei et al., 2014; Kakar et al., 2014; Lambrecht et al., 2022). Unfortunately, a comparison with the healthy contralateral arm is rarely made, whereas it is the standard when investigating recovery after distal radial fractures (DRF). Such studies report recovery in the range of 67%–88% for flexion and 72%–93% for extension (Bot and Ring, 2012). In our 4-D CT evaluation, wrist FE ROM was restored to a mean of 81%, similar to what has been reported after recovery from a DRF. This suggests that the Aptis implant itself does not negatively affect wrist FE and the decrease in wrist FE ROM is more likely to be related to malunion of the radius or other causes such as radiocarpal joint fibrosis (Hattori et al., 2006; Luchetti et al., 2007).

**Figure 10.**
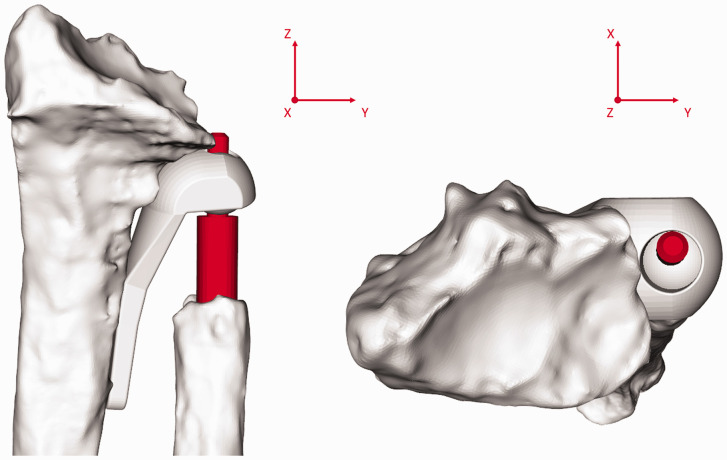
The effect of a deformity of the radius on freedom of movement. The same radius as in Figure 4 is imaged (patient 1). On the right the top of the ulnar stem (orange) is very close to the radial component with the forearm in neutral position. This would impede anticlockwise rotation around the y-axis, which is needed for pronation.

Geometric properties were assessed to identify whether deformation or remodelling of the radius influenced forearm kinematics. Large intra-individual differences were observed. However, no statistical correlation with the kinematic parameters was found. Consideration of corrective surgery of the radius before DRUJ arthroplasty remains important, as a severe deformity of the radius can restrict movements within the range provided by the Aptis implant (Figure 10); specifically, translation and rotation along and around the ulnar stem and angulation around the polyethylene sphere. Consequently, interference between the ulnar stem and the radial component may occur. Inaccuracy of implant placement and the imposition of a different forearm rotation axis may also cause interference.

The number of patient cases in this study was small, which can be considered a limitation. Owing to a lack of similar studies it was not possible to reliably determine an effect size and calculate the sample size for appropriate statistical power. However, the within- individual design of this study makes it inherently more powerful than the more common between-individual designs (Kirk, 2009; Maxwell et al., 2017). Even though a larger sample size would have been preferable, we feel that the results clearly reflect the ability of the Aptis implant to restore healthy forearm kinematics. The lack of preoperative imaging can also be considered a limitation, as it could have linked geometric differences to either the initial radial fracture or remodelling after arthroplasty.

With the quantification of the kinematic and geometric differences we describe, new questions arise. Do these differences lead to complications and even implant failure? If so, how do these complications develop and how can they be prevented? An initial step in answering these questions has been taken, as the present study was carried out in tandem with another of the clinical implications of the arthroplasty procedure (Stougie et al., 2023). Further biomechanical research is needed to confirm the relation between the mismatch in kinematics and complications related to mechanical failure of the implant or periprosthetic fractures.
